# Expression of spindle assembly checkpoint proteins BubR1 and Mad2 expression as potential biomarkers of malignant transformation of oral leukoplakia: an observational cohort study

**DOI:** 10.4317/medoral.24511

**Published:** 2021-10-27

**Authors:** Luís Monteiro, Patrícia Silva, Leonor Delgado, Barbas Amaral, Fernanda Garcês, Filomena Salazar, José Júlio Pacheco, Carlos Lopes, Hassan Bousbaa, Saman Warnakulasuriya

**Affiliations:** 1 UNIPRO – Oral Pathology and Rehabilitation Research Unit, University Institute of Health Sciences (IUCS), CESPU, CRL, Gandra, Portugal; 2 Medicine and Oral Surgery Department, Instituto Universitário de Ciências da Saúde, Gandra, Portugal; 3 Stomatology Department, Hospital de Santo António, Oporto Hospitalar Centre, Porto, Portugal; 4 Molecular Pathology and Immunology Department, Institute of Biomedical Sciences Abel Salazar (ICBAS), Porto University, Porto, Portugal; 5 Centro Interdisciplinar de Investigação Marinha e Ambiental (CIIMAR/CIMAR), Universidade do Porto, Porto, Portugal; 6 Faculty of Dentistry, Oral and Craniofacial Sciences, King’s College London, the WHO Collaborating Centre for Oral Cancer, London, United Kingdom

## Abstract

**Background:**

The Spindle Assembly Checkpoint (SAC) is a surveillance mechanism essential to ensure the accuracy of chromosome segregation during mitosis. Our aim was to evaluate the expression of SAC proteins in oral carcinogenesis, and to assess their potential in predicting malignant transformation of oral leukoplakia.

**Material and Methods:**

We analysed the immunoexpression of BubR1, Mad2, Bub3, and Spindly proteins in 64 oral biopsies from 52 oral leukoplakias and 12 normal tissues. Univariate and multivariate analysis were performed to evaluate predictive factors for malignant transformation (MT).

**Results:**

We observed that BubR1 and Mad2 were more highly expressed in high dysplasia grade lesions than in low grade or normal tissues (*P*<0.05). High expression of Spindly was significantly correlated with a high Ki-67 score (*P*=0.004). Six (11.5%) oral leukoplakias underwent malignant transformation. In univariate analysis, the binary dysplasia grade (high grade) (*P*<0.001) was associated with a higher risk of malignant transformation as well as high BubR1 (*P*<0.001) and high Mad2 (*P*=0.013) expression. In multivariate analysis, high expression of BubR1 and Mad2 when combined showed an increased risk for malignant transformation (*P*=0.013; HR of 4.6, 95% CI of 1.4-15.1).

**Conclusions:**

Our findings reveal that BubR1 and Mad2 were associated with an increased risk for malignant transformation independently of histological grade and could be potential and useful predictive risk markers of malignant transformation in oral leukoplakias.

** Key words:**BubR1, Mad2, Spindly, Bub3, Oral Leukoplakia, epithelial dysplasia, Oral squamous cell carcinoma.

## Introduction

Cancers of lip and oral cavity are within the 10th most common cancers in Europe, with nearly 24,063 estimated deaths in 2018 (1). Almost 90% of oral malignant neoplasms correspond to oral squamous cell carcinomas (OSCC) and only approximately 50% of the patients survive at 5 years of follow-up (1,2).

Interestingly, the majority of the oral cancers in white Caucasoid populations are preceded by mostly white and red patches of oral mucosa, that represent a risk for developing oral cancer, referred to as oral potentially malignant disorders (OPMD). These disorders are defined by the World Health Organization (WHO) Collaborating Centre for Oral Cancer (2020) as lesions or conditions that carry an increased risk for oral cancer development, and include leukoplakia, erythroplakia, lichen planus, and oral submucosal fibrosis (3). They represent morphologic alterations of the oral mucosal tissues that could be the result of molecular alterations of the cells as a consequence of exposure to carcinogenic substances such as tobacco or alcohol. These cumulative genetic/molecular alterations may alter the normal tissue to an OPMD or to an oral carcinoma, which represents the most accepted model for oral carcinogenesis (4). Several genetic pathways may be altered in oral carcinogenesis such as proliferation and cell-controlling pathways, apoptosis and immortalization pathways (5-8).

In the last few years, an increasing attention has been paid to BubR1, Mad2 or Cdc20 proteins, key players of the Spindle Assembly Checkpoint (SAC) mechanism that ensures the fidelity of chromosome segregation (9). In normal cycling cells, unattached or improperly attached kinetochores and spindle damage activate the SAC which in turn halts cells in mitosis, providing additional time for kinetochores to establish a correct bipolar attachment to the mitotic spindle (9). This inhibitory SAC activity is mediated by a diffusible mitotic checkpoint complex (MCC) resulting from the assembly of BubR1, Mad2 and Bub3 proteins with Cdc20, a cofactor and activator of the anaphase-promoting complex or cyclosome (APC/C). Thus, preventing the APC/C-Cdc20 interaction arrests cell in mitosis. On the other hand, once all kinetochores have established proper attachments with spindle microtubules, the MCC disassembles, providing APC/C-Cdc20 interaction which results in Securine and Cyclin B degradation allowing cells to exit mitosis (9). Spindly is a mitotic protein also involved in SAC signalling pathway, namely in SAC silencing, besides its role in chromosome alignment (10). Thus, the activation and/or inactivation of SAC depends on the orchestrated activity of several players, including the key MCC proteins. Indeed, BubR1, Mad2 and Cdc20 hold an important prognostic role on OSCC (9,11). We have observed the independent and significant prognostic role of BubR1 abnormal expression in oral carcinomas even in the early stages (9). Though the role of deregulation of SAC proteins BubR1, Mad2, Bub3, and Spindly have been examined in OSCC their role has not been investigated in OL as predictive markers of oral malignant transformation. Our aim was to evaluate the expression of these proteins in a cohort of oral leukoplakia (OL), and to assess their potential value for predicting malignant transformation.

## Material and Methods

An observational retrospective cohort study was developed to examine the white patches of oral cavity submitted to biopsy with a diagnosis of OL, taken between the period of 1995 to 2006, and archived in the Pathology department of the Centro Hospital do Porto, Portugal. The study was undertaken respecting the Declaration of Helsinki and with the approval of the Institutional ethical committee of the hospital (Investigation, Formation and Teaching Department – DEFI; 024/CES/03) and followed the STROBE guidelines.

From pathology databases and from patients’ records we retrieved demographic, clinical and pathological information including patient’s age, gender, site of the lesion, clinical description of lesions, according to Warnakulasuryia *et al* (3), management, and follow-up information with reference to any recurrence and/or malignant transformation. We included a consecutive case-series of oral leukoplakia from several anatomical sites of the oral cavity (ICD 10: C00-06) with a confirmed histopathology diagnosis of either epithelial hyperplasia and hyperkeratosis or with epithelial dysplasia (pathology review excluded any other known disorder). We included also some biopsied healthy normal tissue obtained during the period of study to serve as controls. We excluded cases under 18-year-old age, or cases with a history of previous oral cancer treatment including radiotherapy and/or chemotherapy or cases without any histopathological confirmation.

With new haematoxylin-eosin stained slides, we reviewed the histopathology characteristics of all included cases. The dysplasia grade was reclassified independently by two observers following the binary classification system of Kujan *et al* (12) grouping cases as low grade (LG) or high grade (HG). The discordant cases were reviewed together and a consensus was achieved.

- Immunohistochemistry

The expression of BubR1, Bub3, Mad2, Spindly and Ki-67 proteins was evaluated by immunohistochemistry on 3-μm tissue sections. Briefly, sections were deparaffinized and rehydrated. Heat-induced antigen retrieval was performed at 98°C for 30 minutes, in a solution of citrate buffer 0.01M at pH 6.0 for Bub3, Spindly and Ki-67 and EDTA buffer 0.01M at pH 9.0 for BubR1 and Mad2 according to manufacturers’ instructions. Endogenous peroxidase activity and non-specific binding were blocked and then tissue sections were incubated for 60 minutes with the primary antibodies: mouse monoclonal anti-BubR1 (clone 9, BD Biosciences Pharmingen, Franklin Lakes, NJ, USA), diluted at 1:150, rabbit polyclonal anti-Bub3 (clone EPR5319(2), Abcam, Cambridge, UK), diluted at 1:500, mouse monoclonal anti-Mad2 (clone 48, BD Biosciences Pharmingen, Franklin Lakes, NJ, USA) diluted at 1:75, rabbit polyclonal anti-spindly (HPA044700, Sigma-Aldrich, St. Louis, USA) diluted at 1:500, and mouse monoclonal anti-Ki-67 (clone MIB-1, Dako, Carpinteria, CA, USA) diluted at 1:100. The immunodetection was performed using a peroxidase-labelled indirect polymer (NovoLinkTM Polymer Detection System; Novocastra, Leica Biosystems Newcastle Ltd) and revealed with DAB chromogen (diaminobenzidine) and counterstained with Gill´s Haematoxylin. Negative controls (omitting the primary antibodies) and positive control sections were included in each staining run (normal skin for BubR1 and Mad2, normal testis tissue for Bub3 and Spindly, and normal tonsil tissue for Ki-67).

- Evaluation of immunohistochemical expression

The evaluation of the stained sections was performed independently by two authors blinded to any of the clinical or pathological characteristics of the cases and using a ZEISS AxioLab A1® microscope (Carl Zeiss Microscopy GmbH, Jena, Germany), with a ZEISS Axiocam 105 colour ® and ZEISS Zen2® software for images caption.

Cases were evaluated according to the percentage of stained keratinocytes (extent of staining) [0 (negative, or <10%); 1+ (10%-24%); 2+ (25%-49%); 3+ (50%-74%) and 4+ (>75%)] and by the intensity of staining [0 (absent), 1 (weak), 2 (moderate), and 3 (strong)] (9,11). Discordant cases were revaluated by the same authors to reach a consensus. For statistical data analysis, cut-off values of extent and intensity scores were defined by ROC curve analysis for each marker (using malignant transformation as event). This resulted in the following categorization of low vs high expression: BubR1 and Mad2 extent, 0/1+ vs 2+/3+/4+; Bub3 and Spindly extent, 0/1+/2+ vs 3+/4+; intensity score (BUBR1, MAD2, Spindly, and BUB3), absent/weak/moderate vs strong intensity. Ki-67, evaluated for percentage of stained cells, was dichotomized in low (0/1+) and high (2+/3+/4+) expression.

- Statistical analysis

The software IBM SPSS Statistics version 25.0 (IBM Corporation, NY, US) was used for statistical analysis. Possible associations between the variables were analysed using the Kruskal-Wallis one-way ANOVA test with pairwise multiple comparisons (with bonferroni adjustment when applicable). The correlation between proliferative cell status (evaluated by Ki-67 protein) and the biomarkers was measured by Spearman’s correlation coefficient. We evaluated the influence of variables for the malignant transformation of OPMD by univariate analysis using the Kaplan-Meier method with the log-rank test and then with the use of Cox proportional hazards model for multivariate analysis.

Oral malignant transformation-free survival (MTFS) rate was defined as the time interval between diagnosis of OPMD and the first occurrence of an oral cancer. The level of significance used for the statistical tests was lower than 5%.

## Results

The final sample corresponded to 64 patients, 46 (71.9%) males and 18 (28.1%) females with a mean age of 58.1±16.8 years-old, including also 12 (18.8%) normal oral tissues. Other clinical characteristics of these cases are presented in Table 1. OL were composed by single lesions in 39 cases and multifocal lesions in 4 cases where this information was available (n=43). Considering the binary histological classification system, there were 41(78.8%) low-grade cases and 11(21.2%) high-grade cases. Most of the cases were submitted to scalpel excision biopsy (n=36; 69.2%), 5 (9.6%) cases to laser ablation, and 11 (21.2%) cases had non-surgical management. Patients included in the study were followed-up for a period ranging 2 to 120 months (mean 32.4 ± 29). There were 13 (25%) recurrences and malignant transformation was reported in 6 (11.5%) out of 52 OL. Of these 6 cases with malignant transformation 4 (66.7%) were males. Two (33.3%) cases were localized in tongue, the others affected with one (16.7%) case for each lip mucosa, floor of the mouth, buccal and retromolar-trigone mucosa. Information regarding tobacco and/or alcohol habits was available only for 5 of these patients, where 3 (60%) of them were smokers and 2 (40%) had an alcohol consumption misuse. Three (75%) cases had a previous non-homogeneous OL and one case (25%) presented multifocal lesions among the available cases (n=4) with this information’s.

Next, we evaluated the immunoexpression of BubR1, Mad2, Bub3, Spindly and Ki-67 proteins in the included cases. Some cases were not valid for evaluation of some markers (loss of sufficient tissue area for evaluation; or sections lost during staining) in particular 4 cases for Mad2 and Bub3, 2 cases for Spindly and one case for Ki-67.

- BubR1

**Figure 1:**
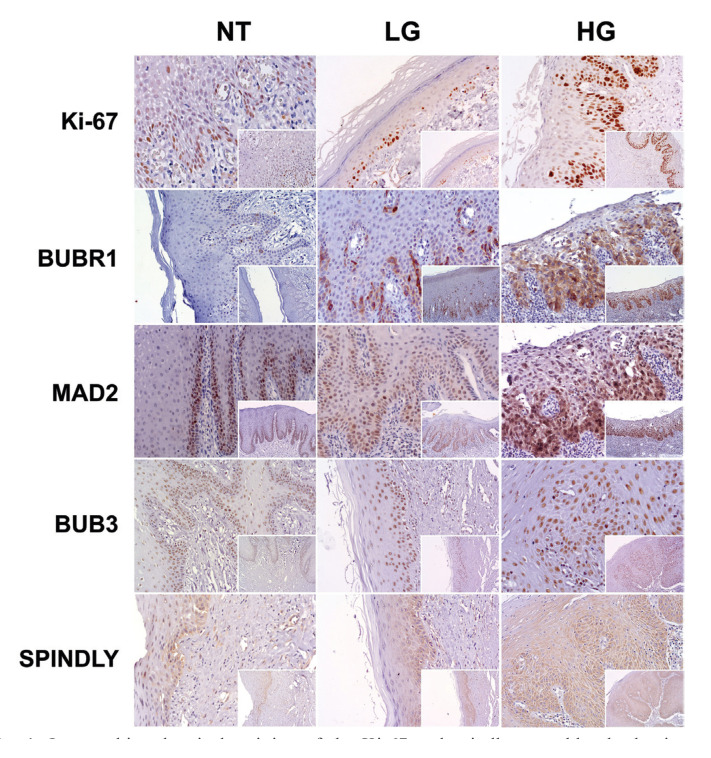
Immunohistochemical staining of the Ki-67 and spindle assembly checkpoint proteins, BUBR1, MAD2, BUB3, and Spindly in normal tissues (NT), low grade (LG) and high grade (HG) oral leukoplakias. Small boxes inside the main photo (magnification ×100), correspond to the original image at magnification ×200. All images in HG group show high expression of the biomarkers, as also for LG group (except for Ki-67) and NT group (except for Ki-67, BubR1 and Spindly).

We observed staining of BubR1, both nuclear and cytoplasmic, in 36 (56.25%) of the cases. We also noted a significant increase of BubR1 extent from focal cellular points of the basal layer to a more continuous staining on one/or two third of the high grade dysplasia cases (Fig. 1). We did not find any case of high extent (upper layers of epithelium) in normal oral mucosa, whereas we observed one case (2.4%) in LG and 4 (36.4%) in HG cases (*P*=0.001). Strong intensity was observed in one case (8.3%) in normal tissues, 16 (39%) in LG, and 11 (72.7%) in HG (*P* =0.007) (Table 1).

**Table 1:**
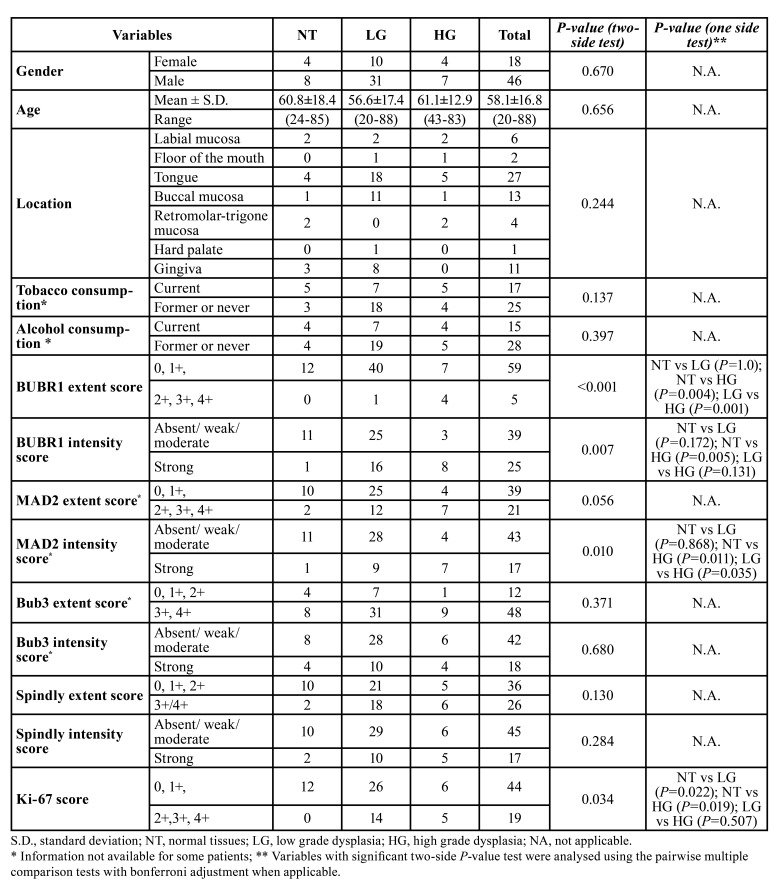
Distribution of the clinical, pathological and biomarkers expression among the groups of patients included in the study.

- Mad2

Expression of Mad2 was observed in the epithelial cells (n=57; 95%) of most included cases. The expression was observed in cell nuclei and cytoplasm, in the basal and suprabasal epithelial layers of NT, extending to the intermediate layers in a diffuse and more continuous pattern in HG lesions (Fig. 1). A high extent score was observed in two cases (16.7%) of NT, 12 (32.4%) of LG, and 7 (63.6%) of HG (*P*=0.05). Regarding the intensity of staining a strong intensity was observed in one case (8.3%) of normal tissues, 9 (24.3%) of LG, and 7 (63.6%) of HG (*P*=0.009) (Table 1).

- Bub3

Most of cases (98.3%) showed nuclear expression of Bub3. The nuclear staining pattern was homogenous and continuous in the epithelial layers of the normal to high grade lesions (Fig. 1). High extent was observed in 8 cases (66.7%) of normal tissues, 31 (81.6%) of LG, and 9 (90%) of HG cases (*P* =0.365). Strong intensity cases corresponded to 4 cases (33.3%) of normal tissues, 10 (26.3%) of LG, and 4 (40%) of HG (*P*=0.676) (Table 1).

- Spindly

Expression of Spindly was observed in the cytoplasm of cells of the most analysed lesions (n=55; 88.7%). The cytoplasmic staining was observed in a continuous pattern in the one third of epithelia and increased in a more diffuse pattern on the other epithelial layers in the high dysplasia cases (Fig. 1). A high extent Spindly score was observed in 2 cases (16.7%) of normal tissues, 18 (46.2%) of LG, and 6 (54.5%) of HG (*P*=0.126). Regarding the intensity of staining, a strong intensity was observed in 2 cases (16.7%) of normal tissues, 10 (25.6%) of LG, and 5 (45.5%) HG cases (*P* =0.278) (Table 1).

- Ki-67 expression and correlation with the other biomarkers

We performed also an evaluation of the proliferative status of the OL using the protein Ki-67. The expression of Ki-67 protein was seen mostly in the cell nucleus of the basal and suprabasal epithelial layers in the NT increasing in a more continuous pattern among the dysplasia cases (Fig. 1). Although some degree of nuclear staining was detected in every case they were classified as high Ki-67 cases in 14 (35%) LG and 5 (45.5%) in HG without any high score case in NT (*P* =0.009) (Table 1). We evaluated the association of this marker as indicative of proliferative activity of the included cases with the expression of the other biomarkers BubR1, Mad2, Bub3 and Spindly. No biomarker was related with proliferative status except for high expression of Spindly with a high Ki-67 score (*P*=0.004) (Table 2).

- Oral Malignant Transformation-free Survival Analysis

**Figure 2:**
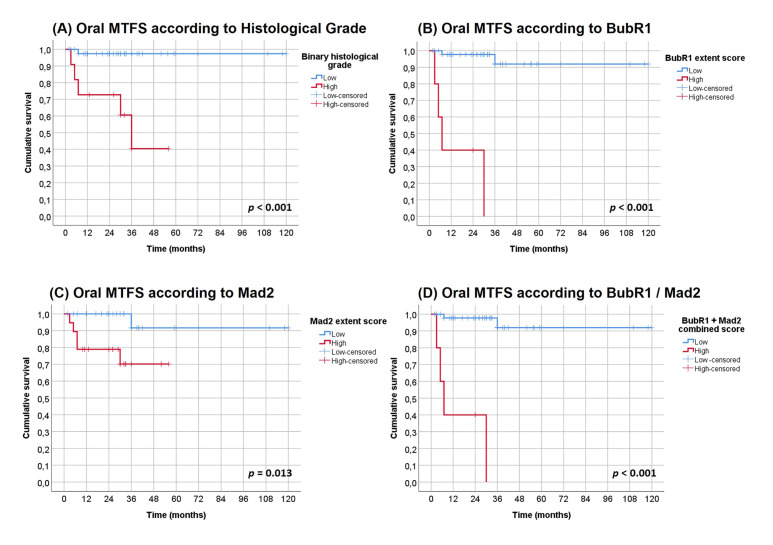
Univariate Kaplan-Meier analysis of oral malignant transformation-free survival (MTFS) in patients with OL. A – MTFS according Histological Grade (*P*<0.001); B – MTFS according BubR1 extent score (*P*<0.001); C –MTFS according MAD2 extent score (*P*=0.013); and D – BubR1 / Mad2 combined extent score (*P*<0.001).

We evaluated the role of the clinical, pathological and immune biomarkers (variables) on malignant transformation of these patients by univariate analysis (Table 3). None of the clinical variables -age, gender, location of the lesion, number (single or multiple) and clinical type of the lesion (homogeneous or non-homogeneous), and management - were related to MTFS. The binary dysplasia grade (high grade) was associated with a higher risk of MTFS (*P*<0.001). Regarding the biomarkers under evaluation, high BubR1 expression (extent score) (*P*<0.001) and high Mad2 expression (extent score) (*P* =0.013) revealed significant *p-value*s for MTFS (Fig. 2).

**Table 2:**
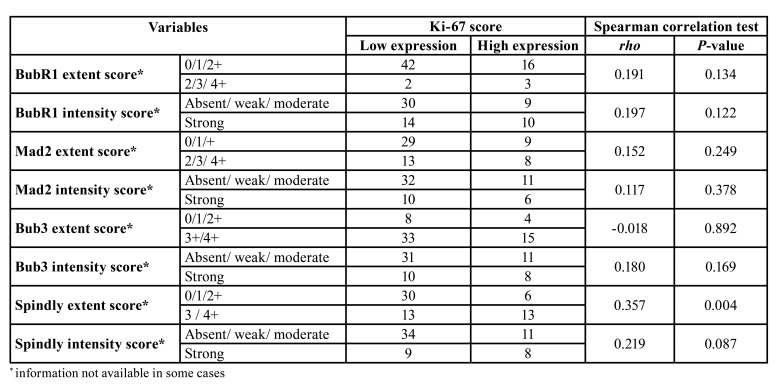
Correlation between proliferative status (assessed by Ki-67 expression) and the other biomarkers.

**Table 3:**
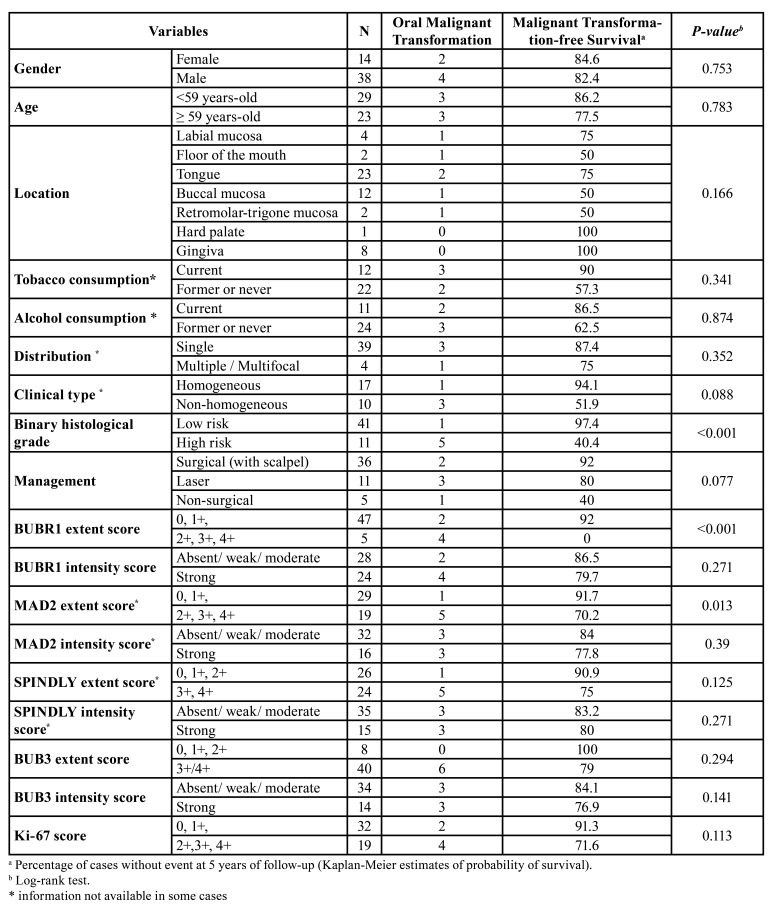
Univariable analysis of the influence of variables in the oral malignant transformation survival rate using Kaplan-Meier curves.

These data enabled us to construct a combined score using both BubR1 and MAD2 extent scores. Using Kaplan-Meier curves we observed a significant effect between patients with high expression of both markers comparing with cases showing low expression of one or both biomarkers (*P* <0.001) (Fig. 2).

Using the Cox regression method including the combined score of Bubr1 and Mad2 and also the binary dysplasia grade we observed an independent predictive risk value only for the combined BubR1 and Mad2 extent score (*P*=0.013; HR of 4.6, 95% CI of 1.4-15.1) (Table 4).

**Table 4:**
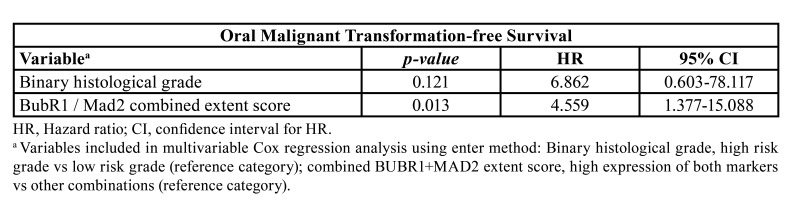
Multivariable analysis of survivals on variables with significant effect in univariable analysis.

## Discussion

Patients diagnosed with OL carry a substantially elevated risk of later developing oral cancer (8). A recent US study reported that OL is associated with a 40.8-fold increased risk of OSCC and a 5-year absolute risk of 3.3% (13).

Research on biomarkers related with malignant transformation of oral leukoplakia and other OPMDs, especially on a longitudinal analysis, are meagre and with little translational evidence of their usefulness (6,7,14,15). Spindle Assembly Checkpoint (SAC) proteins have been described as involved in carcinogenesis of several cancers (both squamous and non-squamous) and were recently described as associated with the aggressiveness and poor tumour prognosis (9,11,16,17). In this study, we analysed the relevance of SAC proteins BubR1, Mad2, Bub3 and Spindly in oral carcinogenesis process, and also to evaluate their potential as predictive biomarkers of malignant transformation. Overall, we found an increasing expression trend of all markers during the different steps of oral carcinogenesis, from normal to hyperplasia to epithelial dysplasia. Notably, BubR1, Mad2 and Ki-67 showed low expression in normal tissue but their expression significantly increased in the presence of dysplasia. This increasing expression was previously reported for BubR1 in oral premalignant lesions by Hsieh *et al* (18) and Sravya *et al* (19), suggesting that the deregulation of SAC is an early and important event in premalignancy. To the best of our knowledge, a significantly increased expression of Mad2 proteins, as reported in the present study, has not been described before. Deregulation of these proteins reflects an inappropriate SAC activity in dividing epithelial cells, which may sustain chromosome instability thereby promoting cancer progression and aggressiveness (16). Effectively, deregulated SAC protein expression, such as the one reported here for BubR1 and Mad2, could be advantageous for abnormal cell proliferation, as a consequence of deficient SAC signaling, unable to ensure efficient control of metaphase to anaphase transition and to avoid chromosome missegregation, thereby fueling chromosome instability. Under these conditions, these cells accumulate aberrant chromosome contents, known to promote malignant transformation and also confer subsequent resistance to treatment.

Also, an association of SAC signalling proteins with the induction of proteins involved in invasion and metastasis such as matrix metallopeptidases (MMPs) have been reported. Chou *et al* (20) have described (in an *in vitro* study) that the down-regulation of BubR1 significantly decreases the activities of MMP-2 and MMP-9 thereby decreasing cellular invasion.

As expected, the high risk OL (classified by binary grade) was related with malignant transformation in univariate analysis. The histological assessment of dysplasia has been the most applied clinic-pathological predictive factor for the risk of oral malignant transformation on OL and reported as an important predictive variable (21-23). However, classical dysplasia grading systems have been criticized as subjective, poorly reproducible, and unpredicTable for clinical management (22). We therefore decided to use the binary histological grade system to classify cases into low risk and high risk cases. Kujan *et al* (12) reported the merits of applying this binary system with less subjectivity to classify dysplasia grade of OPMD. This could be a simple system for routine clinical use as probably low histologic grade cases (without dysplasia or mild dysplasia) would not need any radical or invasive treatment. Nevertheless, dysplasia alone may be insufficient in many cases for malignant transformation as there are cases without or with mild dysplasia that could progress to oral cancer (24) and oral cancers can arise from non-dysplastic OL (13).

Although several studies have addressed the role of biomarkers for the prediction of oral malignant transformation, only a few of them (eg, p53, podoplanin, loss of heterozygosity, or DNA ploidy) have shown significance in longitudinal studies, lacking translational evidence for biomarkers to be applied for clinical routine use (15). We report here a significant role of BubR1 and Mad2 expression in the prediction of malignant transformation of OL. Using a combined score, we demonstrated a significantly predictive role for these two biomarkers in MT in our cohort of patients. Based on these findings we propose that additionally to histological grade classification, the evaluation of BubR1 and Mad2 expression could be useful in OL prognostication. Although the prognostic role of these biomarkers in premalignancy has not been previous reported, BubR1 has shown to be valid for prognostication of several cancers including OSCC (9,24-26). Although, Ki-67 showed an increased expression from NT to LG and HG, we could not find a significant association of this protein indicative of the risk of malignant transformation. Our data are consistent with Zhang *et al* (27) who could not find significance of Ki-67 when performing multivariate analysis in a cohort of patients with OL. A recent systematic review failed to identify any studies showing significant associations of Ki 67 with prognosis by multivariate studies (15).

The expression and especially the increasing expression of these markers during the natural history, particularly during advanced stages (such as high grade OL) suggests that these proteins could be regarded as targets for molecular therapies directed against oral cancer genesis. Recently, we and others have targeted some of these proteins such as BubR1 and Spindly or other SAC proteins for testing new therapeutic approaches *in vitro* or in an adjunctive way of chemotherapeutic drugs (28,29). For instance, it was reported that a reduction on BubR1 levels induces an increase in tumor cell’s sensitivity to clinical relevant doses of paclitaxel (30), resulting from severe chromosome segregation defects.

While the present work presents some limitations such as the small number of cases, the short follow-up time of some included cases, and the retrospective nature of the study with missing clinical information in few cases, we provide data in a longitudinal way with follow-up time to perform a multivariate analysis of cancer-occurrence free survival and to report, for the first time, the potential use of BubR1 and Mad2 that could predict malignant transformation in OL.

We conclude that the SAC proteins BubR1 and Mad2 exhibit an increasing expression from normal tissues, low grade OL to high grade OL, highlighting their potential probable role in oral cancer development. BubR1 and Mad2 could be useful predictive biomarkers of malignant transformation of oral leukoplakia.
